# The Mechanism by Which Safflower Yellow Decreases Body Fat Mass and Improves Insulin Sensitivity in HFD-Induced Obese Mice

**DOI:** 10.3389/fphar.2016.00127

**Published:** 2016-05-23

**Authors:** Huijuan Zhu, Xiangqing Wang, Hui Pan, Yufei Dai, Naishi Li, Linjie Wang, Hongbo Yang, Fengying Gong

**Affiliations:** Key Laboratory of Endocrinology of National Health and Family Planning Commission, Department of Endocrinology, Peking Union Medical College Hospital, Chinese Academy of Medical ScienceBeijing, China

**Keywords:** safflower yellow (SY), obesity, insulin sensitivity, insulin signaling pathway, PGC1α

## Abstract

**Objectives:** Safflower yellow (SY) is the main effective ingredient of Carthamus tinctorius L. It has been reported that SY plays an important role in anti-inflammation, anti-platelet aggregation, and inhibiting thrombus formation. In present study, we try to investigate the effects of SY on body weight, body fat mass, insulin sensitivity in high fat diet (HFD)-induced obese mice.

**Methods:** HFD-induced obese male ICR mice were intraperitoneally injected with SY (120 mg kg^−1^) daily. Eight weeks later, intraperitoneal insulin tolerance test (IPITT), and intraperitoneal glucose tolerance test (IPGTT) were performed, and body weight, body fat mass, serum insulin levels were measured. The expression of glucose and lipid metabolic related genes in white adipose tissue (WAT) were determined by RT-qPCR and western blot technologies.

**Results:** The administration obese mice with SY significantly reduced the body fat mass of HFD-induced obese mice (*P* < 0.05). IPITT test showed that the insulin sensitivity of SY treated obese mice were evidently improved. The mRNA levels of insulin signaling pathway related genes including insulin receptor substrate 1(IRS1), PKB protein kinase (AKT), glycogen synthase kinase 3β (GSK3β) and forkhead box protein O1(FOXO1) in mesenteric WAT of SY treated mice were significantly increased to 1.9- , 2.8- , 3.3- , and 5.9-folds of that in HFD-induced control obese mice, respectively (*P* < 0.05). The protein levels of AKT and GSK3β were also significantly increased to 3.0 and 5.2-folds of that in HFD-induced control obese mice, respectively (*P* < 0.05). Meanwhile, both the mRNA and protein levels of peroxisome proliferator-activated receptorgamma coactivator 1α (PGC1α) in inguinal subcutaneous WAT of SY group were notably increased to 2.5 and 3.0-folds of that in HFD-induced control obese mice (*P* < 0.05).

**Conclusions:** SY significantly reduce the body fat mass, fasting blood glucose and increase insulin sensitivity of HFD-induced obese mice. The possible mechanism is to promote the browning of subcutaneous WAT and activate the IRS1/AKT/GSK3β pathway in visceral WAT. Our study provides an important experimental evidence for developing SY as a potential anti-obesity and anti-diabetic drug.

## Introduction

Obesity is presented as the energy metabolism imbalance with low energy expenditure and high energy uptake, which leads to excessive and abnormal accumulation of fat in our body. It has become a worldwide public health problem due to its strong relationship with diabetes, dyslipidemia, hypertension, coronary heart disease, stroke, sleep apnea, and several kinds of tumors. The costs of treatments for obesity and its related implications have become serious economic burden for health improvement all over the world.

Therefore, weight loss drug development has attracted the attention of researchers around the world. There are two kinds of anti-obesity medications in current or recent medical market. One is the inhibitors of fat absorption, such as orlistat. Another is the modifiers of central nervous system neurotransmission of norepinephrine, dopamine and serotonin, such as sibutrimin (Padwal et al., 2003). The function of these drugs improving insulin resistance is often dependent on the weight loss, rather than on the direct effect to the target genes. And these anti-obesity drugs have many adverse events that outweigh the modest benefits of treatment (Ioannides-Demos et al., 2011). Orlistat can lead to obligate increases in undigested stool triglycerides which may cause oily spotting, flatus with discharge, fecal urgency, fatty oily stool, increased defecation, fecal incontinence, and other considerable gastrointestinal adverse effects (Yanovski and Yanovski, 2014). The central acting drugs sibutrimin and phentermine can cause elevation in heart rate and blood pressure, insomnia, dry mouth, dizziness, tremors, headache, vomiting, and anxiety (Yanovski and Yanovski, 2014). These adverse effects lead to a decline in the life quality of patients and are one reason of their therapy interruption. Traditional Chinese medicine is gradually being paid close attention because of its beneficial features including multi-target effects, rich resources, less side effects and safety. So it is necessary to explore the weight loss drugs or ingredients from traditional Chinese medicine by using modern molecular biological technology.

Safflower yellow (SY), the main effective ingredient of Carthamus tinctorius L, has many different functions, including anti-inflammation, anti-platelet aggregation, inhibiting thrombus formation, anti-oxidation and inhibiting tumor angiogenesis (Liu L. et al., 2012; Liu S. X. et al., 2012; Li et al., 2013; Wang et al., 2014). The parenteral solution, tablets or other dosage forms of Carthamus tinctorius L have been approved by China Food and Drug Administration (CFDA) as an adjunctive therapy for cardiovascular and cerebrovascular diseases. Our previous studies showed that hydroxysafflor yellow A (HSYA), the main active ingredient in SY, could inhibit the proliferation and adipogenesis of murine 3T3-L1 preadipocytes by promoting lipolytic-specific enzyme hormone sensitive lipase (HSL) expression (Zhu et al., 2014). However, it is still unknown whether SY has effects on the body weight and insulin sensitivity *in vivo*. In this study, we try to explore the effects of SY on body weight, body fat mass, insulin sensitivity and its possible tissue targets in high fat diet (HFD)-induced obese mice. Our results showed that SY could reduce body fat mass of HFD-induced obese mice by promoting the browning of subcutaneous white adipose tissue (WAT), and decrease blood glucose levels and increase insulin sensitivity through activating IRS1/AKT/GSK3β insulin signaling pathway in visceral WAT of HFD-induced obese mice.

## Methods

### Animals

Eight-week-old male ICR mice (25~27 g) were obtained from Beijing HFK Bioscience Co., LTD. SY was obtained from Prof. Ming Jin (Department of Pharmacology, Beijing Anzhen Hospital, Capital Medical University) who isolated and purified SY from the aqueous extracts of C. tinctorius L. by macroporous resin-gel column chromatography and determined a purity of 95.4% by HPLC as they described previously (Zang et al., 2008; Song et al., 2013). All mice were randomly divided into standard food (SF) group (*n* = 10) and HFD group (*n* = 25). Eight weeks later, mice fed with HFD were weighed and divided into HFD group (*n* = 13) and SY intervention group (SY group, *n* = 12). All mice were housed in standard cages with access to food and water *ad libitum* in a temperature-controlled room (21–23°C) and under a 12 h dark/light cycle. Mice in SY group were intraperitoneally injected with SY at a concentration of 120 mg kg^−1^ daily, and mice in SF and HFD groups were intraperitoneally injected with saline in equal volume daily. The body weight of mice was measured once a week. After 8 weeks intervention, the intraperitoneal insulin tolerance test (IPITT) and intraperitoneal glucose tolerance test (IPGTT) were performed and mice were killed by cervical vertebra dislocation. Inguinal subcutaneous and visceral WAT including mesenteric, epididymal, and perirenal WAT was dissected, weighted and then frozen immediately in liquid nitrogen for the further analysis. Serum insulin, fasting blood glucose (FBG), uric acid (UA), Lipoprotein (a) [Lp (a)], high-sensitivity C-reactive protein (hsCRP), triglycerides (TG), total cholesterol (TC), free fatty acids (FFA), low density lipoprotein-cholesterol (LDL-c), and high density lipoprotein-cholesterol (HDL-c) of mice were measured by routine automated laboratory methods. All animal experimental protocols were carried out according to the standards of the Guide for the Care and Use of Laboratory Animals and approved by the ethics committee of Peking Union Medical College Hospital.

### Real-time fluorescence quantitative RT-PCR (RT-qPCR) analysis

RT-qPCR was performed with SYBR green fluorescent dye using an ABI7500 PCR system as previously described (Livak and Schmittgen, 2001; Gong et al., 2009). In brief, adipose tissue total RNA was isolated by using E.Z.N.A Total RNA Kit I (Omega Biotek, USA, Lot R6834-01). 0.5 μg of total RNA was used to produce cDNA using an RT-qPCR system including Omniscript RT kit (Qiagen, USA, Lot 205111), Oligo (dT) primer (Promega, capsule, Lot C110A), and RNA enzyme inhibitor (Promega, capsule, Lot N251A). Housekeeping gene *Cyclophilin A* (*PPIA*) was used as internal control to normalize the expression of target genes. The primer sequences of target genes and internal control gene were listed in Table S1. RT-qPCR was duplicated for 2 wells in a final volume of 20 μL. The mRNA expression levels of target genes in arbitrary unit were acquired from the value of the threshold cycle (Ct) of the RT-qPCR as related to that of *PPIA* using the comparative Ct method through the formula 2^−ΔΔCT^. All samples were normalized to the *PPIA* values and the results expressed as fold changes of Ct value relative to control by using the formula 2^−ΔΔCt^ (Gong et al., 2009, 2010).

### Western blot analysis

Total proteins were extracted from adipose tissue samples using a Total Protein Extraction kit (Applygen, Beijing, China) according to the manufacturer's instructions. The protein contents were determined using the Coomassie Protein Kit (Applygen, Beijing, China). Total protein concentration was about 2 μg ul^−1^. The proteins were separated by SDS-PAGE and transferred onto nitrocellulose membranes (Millipore, USA) by using a wet transfer (BIO-RAD, California, USA). The membranes were blocked with 5% non-fat milk diluted using TBST (Jiangchenyuanyuan Biotechnology, Beijing, China) and then incubated with primary antibodies at 4°C overnight. Mouse immunoglobulin G (IgG) anti-β-actin (Sigma, USA), anti-PGC1α (Millipore, USA), and Rabbit IgG anti-AKT (Cell Signaling Technology, USA), anti-GSK3β (Cell Signaling Technology, USA) were used as the primary antibodies (1:1000 to 1:4000 diluted in 5% non-fat milk). The membranes were washed with TBST and then incubated with horseradish peroxidase (HRP)-conjugated goat antibodies (Applygen, China) (1:1000 diluted 5% non-fat milk) at room temperature for 1.5 h. The specific protein bands were visualized by enhanced chemiluminescence (ECL) according to the manufacturer's instructions (Applygen, Beijing, China). The bands were then quantified by Quantity One software (Version 4.6.9, BIO-RAD, California, USA). β-actin was used as an internal control for target proteins analysis.

### Statistical analysis

Results were expressed as mean ± standard error (SE). The C_*t*_ values obtained in RT-qPCR analysis were transformed through the formula 2^−ΔΔCT^ to linear relationship. The density ratio of target protein to reference protein was calculated to evaluate the protein expression differences. Univariate analysis of variance (ANOVA) method was used for data analysis. Dunnett's T3 method was used for two group comparisons depending on the homogeneity of variance. Skewed data were ln-transformed and Mann-Whitney U test was used if data were still not normally distributed. All statistical computations were run on SPSS 13.0 for Windows (SPSS Inc, Chicago, IL, USA), *p* < 0.05 was considered as statistically significant.

## Results

### Effects of SY on body fat mass, biochemical parameters, and insulin sensitivity in HFD induced obese mice

Body weight of mice fed with HFD for 8 weeks were significantly increased and reached to 1.22-folds of mice fed with standard food (SF) (53.8 ± 1.6 g vs. 44.0 ± 1.1 g, *P* < 0.05), implying obese mice model was established successfully (Figure S1). The body weight of obese mice in HFD group was always significantly higher than those in SF group during the period of 8-week SY administration (Figure S2, *P* < 0.05), and it was 126% of that in SF group (Table 1) at the end of administration. However, there was no significant body weight reduction was observed in SY group mice when compared with that in HFD group (Table 1). Meanwhile, the mass of subcutaneous, mesenteric, perirenal, and epididymal fat tissue of obese mice were significantly increased to 5.12, 1.87, 4.48, and 3.47-folds of that in SF group mice (all *P* < 0.05). Interestingly, the fat mass of these locations in SY administration mice was significantly decreased by 42.6, 30.0, 40.2, and 24.8% of that in HFD group mice, respectively, as presented in Table 1, and significant differences were observed in subcutaneous and perirenal adipose tissue (all *P* < 0.05). As shown in Table 1, the total fat mass and the percentage of body weight occupied by the total fat mass of HFD-induced obese mice were significantly increased to 3.36 and 2.53-folds in comparison with that in SF group (all *P* < 0.05). After 8 weeks, the total fat mass and the percentage in SY administration mice was significantly decreased by 33.0 and 29.4% of that in HFD group mice, respectively (*P* < 0.05).

**Table 1:** **Effects of SY on body weight, body fat contents and biochemical parameters in HFD-induced obese mice**.

	**SF (*n* = 10)**	**HFD (*n* = 13)**	**SY (*n* = 12)**
Body weight (g) before administration	44.00±1.06	54.83±2.26a	53.00±2.03
Body weight (g) after administration^#^	44.97±0.90	56.72±2.19^a^	54.36±3.16
Subcutaneous fat mass (g)	0.33±0.11	1.69±0.84^a^	0.97±0.65^b^
Mesenteric fat mass (g)	0.63±0.22	1.18±0.57^a^	0.85±0.40
Perirenal fat mass (g)	0.25±0.07	1.12±0.59^a^	0.67±0.36^b^
Epididymal fat mass (g)	0.64±0.14	2.22±0.85^a^	1.67±0.80
Total fat mass (g)	1.85±0.14	6.21±0.71^a^	4.16±0.58^b^
Percentage of fat mass^*^ (%)	4.70±0.31	11.91±1.01^a^	8.41±0.96^b^
FBG (mmol/L)	5.22±0.75	8.00±0.78^a^	5.83±0.68^b^
UA (μmol/L)	191.90±7.25	195.08±12.85	264.83±24.10^b^
TC (mmol/L)	4.25±0.32	4.90±0.27	4.76±0.30
TG (mmol/L)	0.43±0.05	0.40±0.07	0.50±0.05
HDL-c (mmol/L)	2.23±0.13	2.33±0.12	2.22±0.10
LDL-c (mmol/L)	0.47±0.08	0.54±0.05	0.60±0.05
Lp(a) (mg/L)	4.10±0.53	4.77±0.47	5.67±0.38
hsCRP (mg/L)	0.08±0.01	0.08±0.01	0.09±0.01
FFA (μmol/L)	1287.50±109.83	1129.85±82.66	1201.67±66.08
Insulin (ng/ml)	0.47±0.05	0.52±0.06	0.73±0.11

Values are means ± SE; SF, standard food; HFD, high fat diet; SY, safflower yellow; FBG, fasting blood glucose; UA, uric acid; TC, total cholesterol; TG, triglycerides; HDL-c, high-density lipoprotein cholesterol; LDL-c, low-density lipoprotein cholesterol; Lp(a), lipoprotein (a); hsCRP, high-sensitivity C-reactive protein; FFA, free fatty acids;

# *,body weight after administration for 8 weeks*;

* *, Total fat mass/Body weight*;

a *,compared with SF group, P < 0.05*;

b *,compared with HFD group, P < 0.05*.

The changes of biochemical parameters in obese mice treated with SY for 8 weeks were displayed in Table 1. The obese mice in HFD group had higher FBG levels as compared with that in SF group (8.00 ± 0.78 mmol L^−1^ vs. 5.22±0.75 mmol L^−1^, *P* < 0.05). After administration the mice with SY for 8 weeks, the FBG levels were significantly reduced by 27.1% as compared with that in HFD group (5.83 ± 0.68 mmol L^−1^ vs. 8.00 ± 0.78 mmol L^−1^, *P* < 0.05). Meanwhile, the UA levels of mice in SY group were significantly increased by 35% in comparison with that in HFD group (264.83 ± 24.10 μmol L^−1^ vs. 195.08 ± 12.85 μmol L^−1^, *P* < 0.05). However, there was no significant difference with regard to TC, TG, HDL-c, LDL-c, Lp (a), hsCRP, FFA, and insulin levels in these three groups.

**Figure 1:**
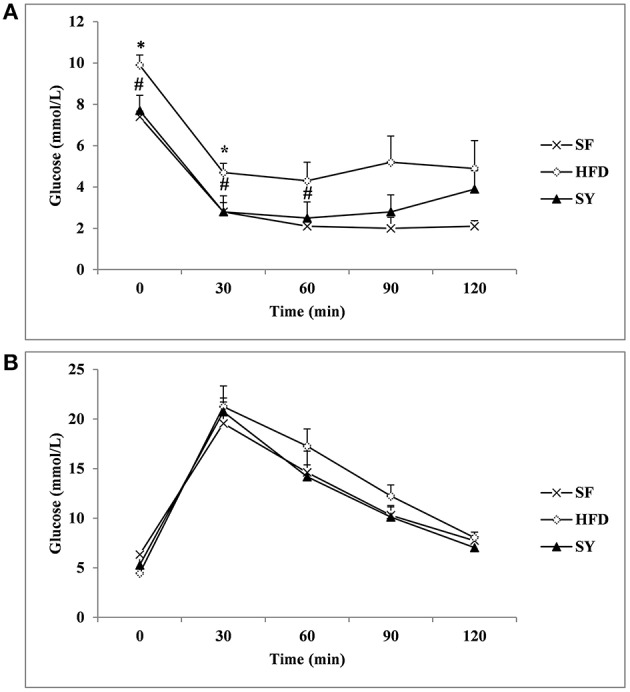
**IPITT and IPGTT in the three groups**. After 8 weeks intervention, the intraperitoneal insulin tolerance test (IPITT) **(A)** and intraperitoneal glucose tolerance test (IPGTT) **(B)** in mice were performed. Glucose levels at 0 (before injection), 30, 60, 90, 120 min were measured.^*^P < 0.05 vs. SF group; ^#^*P* < 0.05 vs. HFD group.

Next, the 2-h IPITT and IPGTT tests were done to explore the effect of SY on glucose metabolism. As presented in Figure 1A, the glucose curve in IPITT tests showed that glucose at every time point in HFD group was increased when compared with those in SF group, and significantly increased by 33.8 and 67.9% at 0, 30 min (*P* < 0.05). After administration obese mice with SY for 8 weeks, the glucose levels at every time point was significantly reduced by 22.2, 40.4, and 41.9% at 0, 30, 60 min in IPITT tests when compared with those in HFD group (*P* < 0.05). However, there was no significant difference of glucose level at every point in IPGTT among the three groups as shown in Figure 1B (*P* > 0.05).

### Effects of SY on the expression of genes involved in insulin signaling pathways in WAT of obese mice

**Figure 2:**
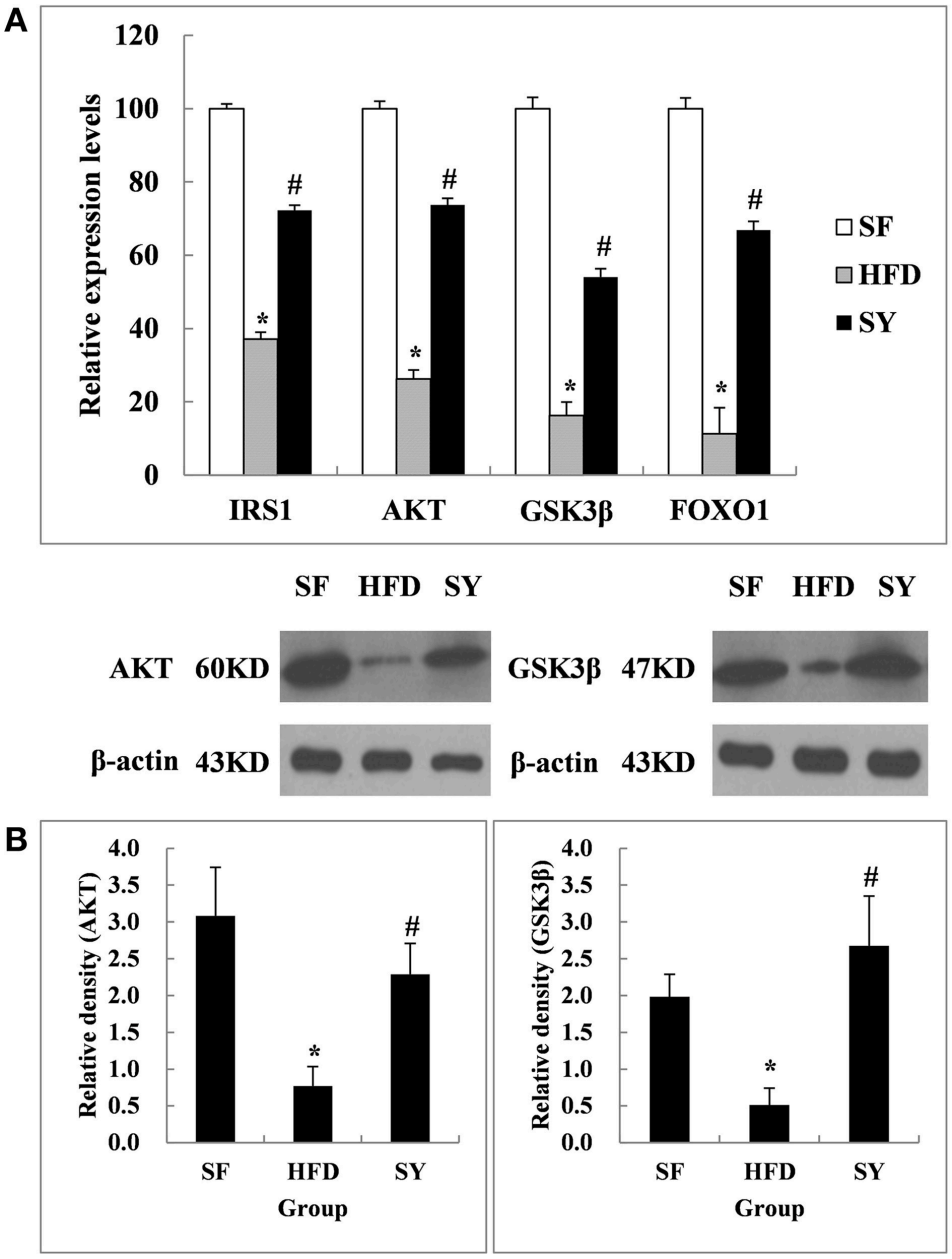
**Effects of SY on the mRNA and protein levels of genes involved in insulin signaling pathways in mesenteric white adipose tissue of obese mice**. **(A)** The mRNA levels of *IRS1, AKT, GSK3*β, and *FOXO1* in mesenteric white adipose tissue of mice in SF, HFD and SY group were determined by RT-qPCR analysis. The RT-qPCR was repeated for two times with *n* = 8 for each group. **(B)** The protein levels of AKT and GSK3β in mesenteric white adipose tissue of mice in SF, HFD, and SY group were determined by western blot analysis. The blots were repeated for three times with *n* = 8 for each group. ^*^P < 0.05 vs. SF group; ^#^*P* < 0.05 vs. HFD group.

In order to explore the tissue targets of SY decreasing fasting blood glucose and increasing insulin sensitivity, the expression of genes involved in insulin signaling pathway including *IRS1, AKT, GSK3*β, and *FOXO1* were investigated. As presented in Figure 2A, the mRNA levels of *IRS1, AKT, GSK3*β, and *FOXO1* in mesenteric WAT of HFD-induced obese mice were significantly decreased by 62.9, 73.8, 83.7, and 88.7% of that in SF mice (*P* < 0.05). After administration obese mice with SY for 8 weeks, the mRNA levels of these genes were notably increased to 1.9-, 2.8-, 3.3-, and 5.9-fold of that in HFD-induced obese mice (*P* < 0.05). In consistent with the changes in transcription levels, the protein levels of AKT and GSK3β in mesenteric WAT of obese mice were also significantly decreased by 75.0 and 74.2%, respectively, in comparison with SF mice (Figure 2B, *P* < 0.05) and notably increased to 3.0- and 5.2-fold of that in HFD-induced obese mice, respectively, after administration these mice with SY for 8 weeks (Figure 2B, *P* < 0.05).

**Figure 3:**
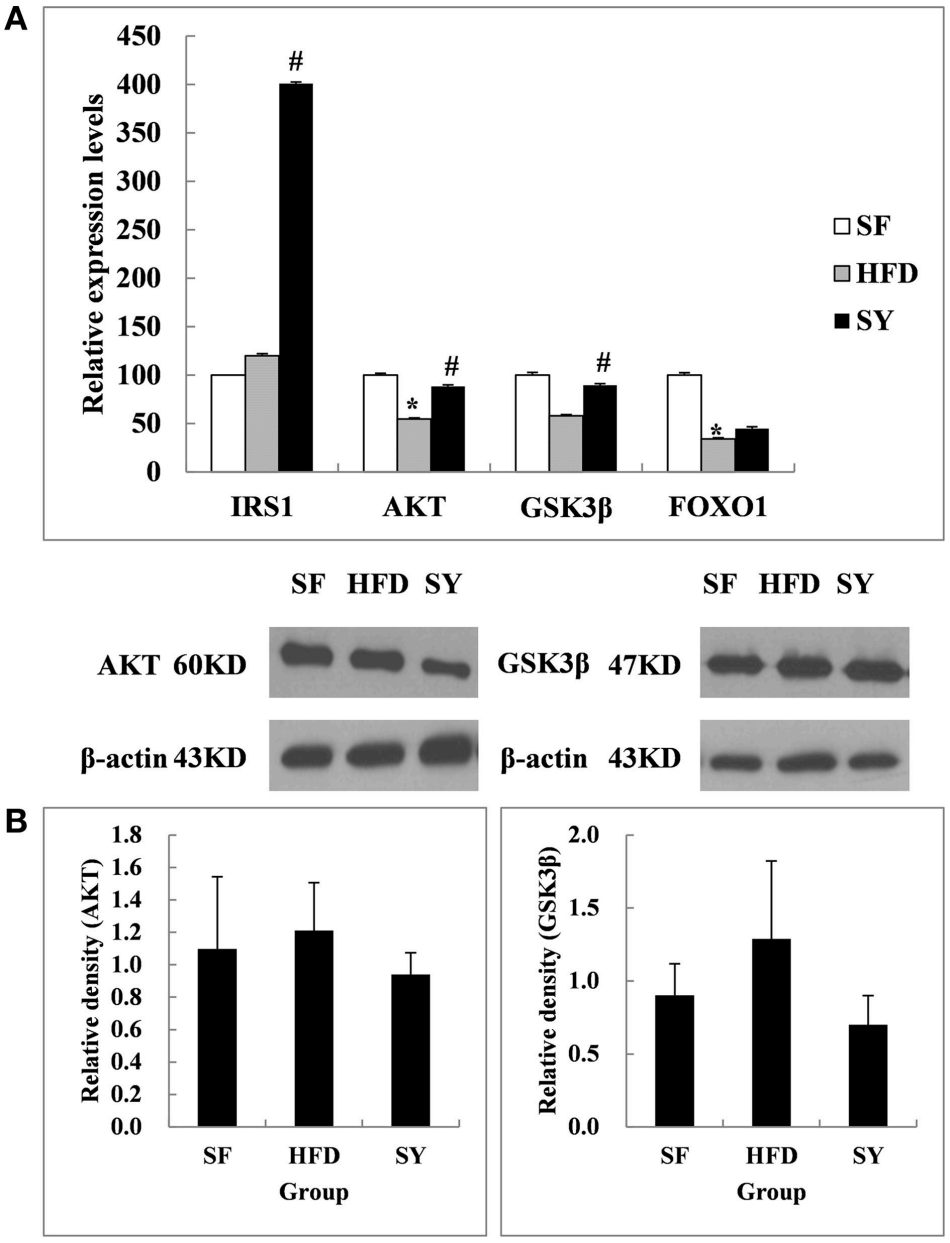
**Effects of SY on the mRNA and protein levels of genes involved in insulin signaling pathways in subcutaneous white adipose tissue of obese mice**. **(A)** The mRNA levels of *IRS1, AKT, GSK3*β, and *FOXO1* in subcutaneous white adipose tissue of mice in SF, HFD, and SY group were determined by RT-qPCR analysis. The RT-qPCR was repeated for two times with *n* = 8 for each group. **(B)** The protein levels of AKT and GSK3β in subcutaneous white adipose tissue of mice in SF, HFD, and SY group were determined by western blot analysis. The blots were repeated for three times with *n* = 8 for each group. ^*^P < 0.05 vs. SF group; ^#^*P* < 0.05 vs. HFD group.

As what we did in the above mesenteric WAT, inguinal subcutaneous WAT was also used to investigate the changes of the insulin signaling pathway related genes after SY administration. As shown in Figure 3A, the mRNA levels of *AKT* and *FOXO1* in inguinal subcutaneous WAT of HFD-induced obese mice were significantly decreased by 45.3 and 65.9% of that in SF mice (*P* < 0.05), but the mRNA levels of *IRS1* and *GSK3*β were not significantly changed (*P* > 0.05). After administration obese mice with SY for 8 weeks, the mRNA levels of *AKT, GSK3*β, and *IRS1* were significantly increased to 1.6-, 1.5-, and 3.3-fold of that in HFD-induced obese mice (*P* < 0.05), but the mRNA levels of *FOXO1* were not significantly changed (*P* > 0.05). However, inconsistent with the changes in transcription level, the protein levels of AKT and GSK3β in subcutaneous WAT had no significant changes among the three groups as presented in Figure 3B (*P* > 0.05).

### Effects of SY on the expression of genes involved in the browning of WAT in adipose tissue

**Figure 4:**
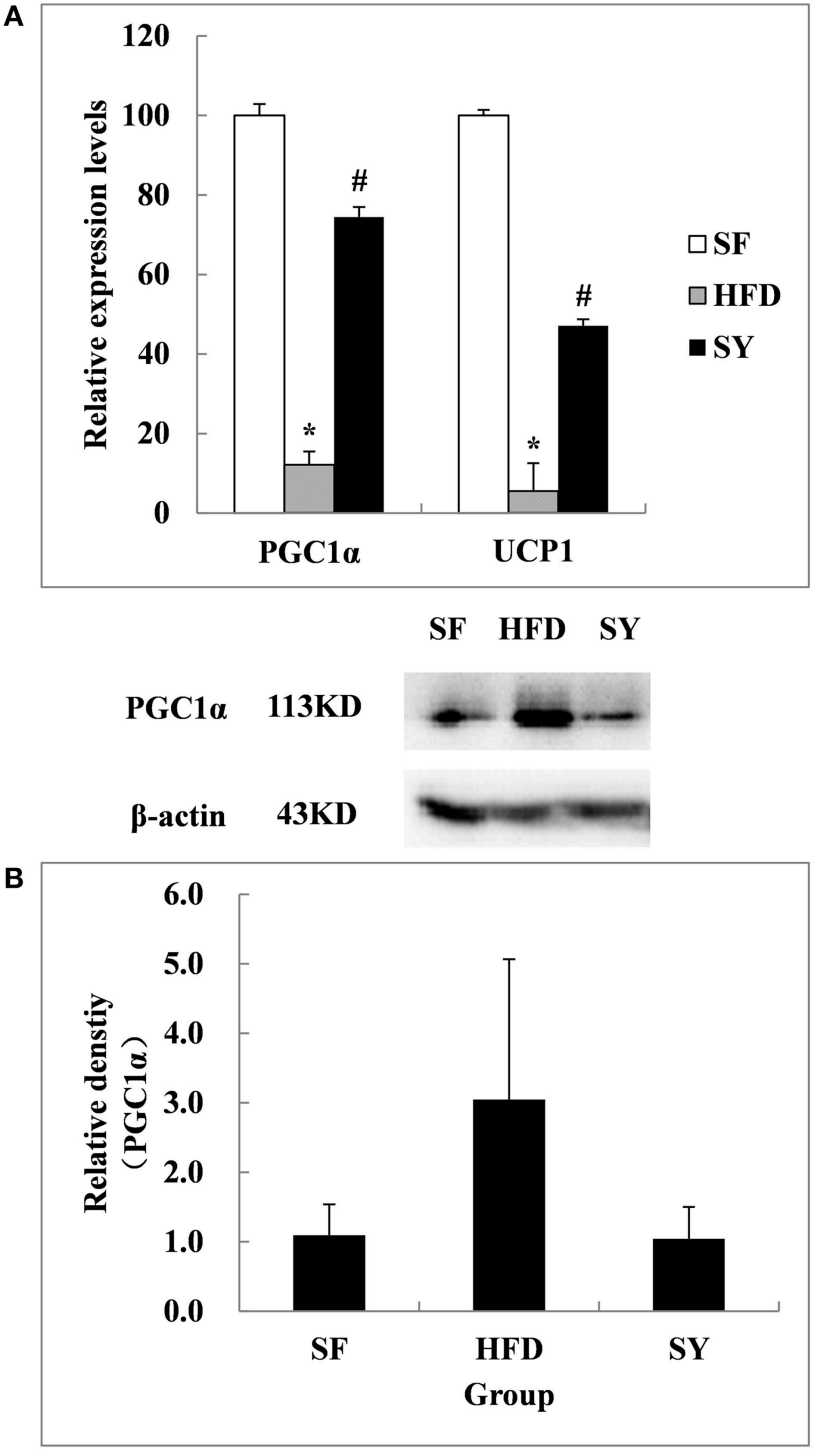
**Effects of SY on the mRNA and protein levels of genes involved in the browning of white adipose tissue in mesenteric white adipose tissue of obese mice**. **(A)** The mRNA levels of *PGC1*α and *UCP1* in mesenteric white adipose tissue of mice in SF, HFD, and SY group were determined by RT-qPCR analysis. The RT-qPCR was repeated for two times with *n* = 8 for each group. **(B)** The protein levels of PGC1α in mesenteric white adipose tissue of mice in SF, HFD, and SY group were determined by western blot analysis. The blots were repeated for three times with *n* = 8 for each group. ^*^P < 0.05 vs. SF group; ^#^*P* < 0.05 vs. HFD group.

In order to explore the tissue targets of SY decreasing fat content, the expression of genes involved in the browning of WAT including *PGC1*α*, UCP1, FNDC5, PRDM16*, and *CIDEA* were investigated. As presented in Figure 4A, the mRNA levels of *PGC1*α and *UCP1* in mesenteric WAT of HFD-induced obese mice were significantly decreased by 87.9 and 94.5% of that in SF mice (*P* < 0.05). After administration obese mice with SY for 8 weeks, the mRNA levels of these genes were significantly increased to 6.2- and 8.6-fold of that in HFD-induced obese mice (*P* < 0.05). However, inconsistent with the changes in transcription levels, the protein levels of PGC1α and UCP1 in mesenteric WAT had no significantly differences among the three groups as presented in Figure 4B (*P* > 0.05).

**Figure 5:**
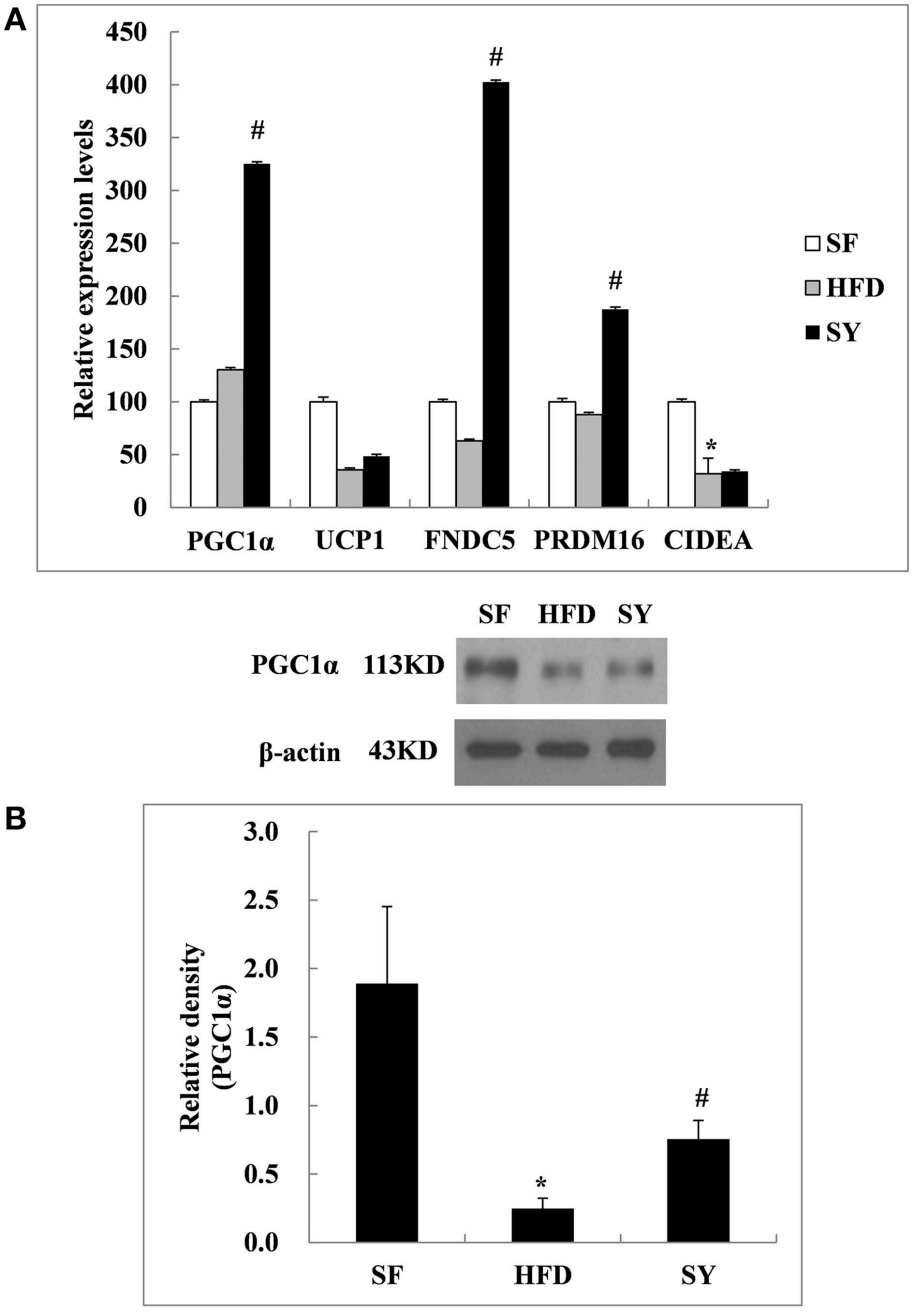
**Effects of SY on the mRNA and protein levels of genes involved in the browning of white adipose tissue in subcutaneous white adipose tissue of obese mice**. **(A)** The mRNA levels of *PGC1*α*, UCP1, FNDC5, PRDM16*, and *CIDEA* in subcutaneous white adipose tissue of mice in SF, HFD, and SY group were determined by RT-qPCR analysis. The RT-qPCR was repeated for two times with *n* = 8 for each group. **(B)** The protein levels of PGC1α in subcutaneous white adipose tissue of mice in SF, HFD, and SY group were determined by western blot analysis. The blots were repeated for three times with *n* = 8 for each group. ^*^P < 0.05 vs. SF group; ^#^*P* < 0.05 vs. HFD group.

As what we did in the above mesenteric WAT, inguinal subcutaneous WAT was also used to investigate the changes of the above five genes after SY administration. As shown in Figure 5A, the mRNA levels of *PGC1*α*, FNDC5*, and *PRDM16* in inguinal subcutaneous WAT of HFD-induced obese mice were not significantly changed as compared with SF mice (*P* > 0.05). After administration obese mice with SY for 8 weeks, the mRNA levels of these genes were significantly increased to 2.5- , 6.4- , and 2.1-folds of that in HFD-induced obese mice (*P* < 0.05). In agreement with the changes in transcription levels, the protein levels of PGC1α in subcutaneous WAT of obese mice were significantly decreased by 87.0% in comparison with SF mice (*P* < 0.05), while increased to 3.0-fold of that in HFD-induced obese mice after SY administration as shown in Figure 5B (*P* < 0.05). However, the mRNA levels of *UCP1* in inguinal subcutaneous WAT had no significant difference among the three groups. Additionally, the mRNA levels of *CIDEA*, a characteristic gene of classical brown adipose tissue, in inguinal subcutaneous WAT of HFD-induced obese mice were significantly decreased by 68.2% of that in SF mice (*P* < 0.05). However, the mRNA levels of this gene were not significantly changed after administration these mice with SY for 8 weeks as shown in Figure 5A (*P* > 0.05).

## Discussion

SY is the main ingredient of Carthamus tinctorius L which is one kind of the safflower plant. It contains flavonols, chalcones, alkaloids and other chemical compositions. SY has a structure of chalcone and is a kind of water-soluble composition. SY has been widely used in the treatment of cardiovascular and cerebrovascular ischemic disease in clinical practice.

At present, many pharmacological effects of SY have already been proved, including anti-platelet aggregation, anti-inflammation, anti-oxidative stress, anti-fibrosis and so on. Our study for the first time demonstrated that SY could reduce the body fat mass, fasting blood glucose and increase insulin sensitivity of HFD-induced obese mice.

Insulin signaling pathway is a key element for insulin sensitivity. Insulin binds to the insulin receptor to cause a series of protein phosphorylation cascade through IRS/phosphatidylinositol 3 kinase (PI3K)/AKT pathway, and eventually lead to glucose uptake and gene expression related with glucose metabolism. On the condition of obesity and its related metabolic abnormalities, the impaired insulin signaling pathway usually leads to insulin resistance.

AKT (also known as PKB), which is a serine / threonine specific protein kinase (Gonzalez and Mcgraw, 2009) plays a crucial role in the insulin signaling pathway (Mora et al., 2004). The main function of AKT pathway is promoting glucose uptake by muscle and adipose tissue and increasing hepatic glycogen synthesis. Meanwhile, it also inhibited glucose release from hepatocytes and gluconeogenesis (Steinberg and Kemp, 2009). The enhancement of AKT pathway can improve insulin sensitivity through affecting GSK3β and other downstream factors. In the present study, the mRNA and protein expression levels of genes involved in AKT pathway in visceral WAT of obese mice were significantly decreased. This phenomenon represented the impairment of insulin signaling pathway in visceral WAT. After SY intervention for 8 weeks, the mRNA and protein expression levels of AKT and GSK3β were significantly increased. However, the similar phenomenon was not observed in the subcutaneous WAT. These results suggested that SY may play a different role in adipose tissue from different locations. SY decreased FBG and improved insulin sensitivity mainly through the recovery of the function of insulin signaling pathway in visceral WAT, rather than in subcutaneous WAT. In agreement with our results, Li et al. (2014) found that insulin resistance induced by liquid fructose in adipose tissue of rat could be reduced through increasing the protein expression levels of AKT and activating its signaling pathway. Liu and Wang et al also found that SY could increase the protein expression levels of AKT in myocardial cells (Liu S. X. et al., 2012) and cardiac muscle tissue (Wang, 2008). All these findings, together with our results, suggested that SY improved insulin sensitivity and decreased blood glucose level through its effects on the IRS1/AKT/GSK3β pathway in visceral WAT.

Obesity usually represents as weight gain, but its most important characteristic is the abnormal increase of body fat contents. Reducing body fat content could effectively improve insulin sensitivity and decrease blood glucose. In the present study, we observed the decrease of fat content and percentage in SY-treated obese mice. So the effects of SY on the reduction of FBG and the improvement of insulin sensitivity may also be the subsequent effects of the decrease of body fat contents. It was found that the glucose uptake rate by insulin stimulating was negatively correlated with abdominal fat content in adipose tissue of patients with abdominal obesity (Virtanen et al., 2005). Galgani et al. (2013) also found high expression of adipose gene (*adp/WDTC1*) was associated with adipose tissue reduction and glucose utilization increase in human adipose tissue, while inhibition of lipid formation was found to be related with *adp* over expression in mice. Consistently, studies performed by Lucas et al. (2012) and Choi et al. (2007) also showed that insulin sensitivity improved while fat content decreased in obese mice. Therefore, apart from the stimulatory role of SY on the insulin signaling pathway, the effects of SY improving insulin sensitivity and reducing blood glucose were supposed to be partly associated with the reduction of body fat contents in HFD-induced obese mice.

There are two traditional types of adipose tissue with different functions, white and brown adipose tissue. In recent years, they were considered to exist together and could be transformed into each other under certain conditions including cold stimulation and β3 adrenergic receptor agonist treatment (Collins et al., 1997; Ghorbani and Himms-Hagen, 1997; Ghorbani et al., 1997; Giordano et al., 1998; Granneman et al., 2005; Vitali et al., 2012). The transformation from WAT to brown adipose tissue was called the browning of WAT (Barbatelli et al., 2010; Vitali et al., 2012). PGC1α, a transcriptional coactivator, is involved in this process. It was considered as a key regulator and could up-regulate the expression of FNDC5 to promote the browning of WAT (Bostrom et al., 2012). PRDM16 (Seale et al., 2011) was also considered to be another important regulator involved in this process. In the present study, we for the first time found that SY could reduce the fat mass of obese mice. Further research demonstrated that the mRNA expression levels of *PGC1*α*, FNDC5, PRDM16*, and the protein expression level of PGC1α in the subcutaneous WAT were significantly increased. All these results implied that SY may reduce the fat mass of obese mice by promoting the browning of the subcutaneous WAT. However, the protein expression level of PGC1α had no significant difference in mesenteric WAT after SY administration. It was suggested that the mechanism of SY promoting the browning of WAT may be different in the adipose tissue from different locations. Similar to our results, Haruya (Ohno et al., 2012) et al found that only subcutaneous WAT could be induced to browning by PPARγ agonists through the activation of the PRDM16 pathway. Seale et al. (2011) and many other studies (Wu et al., 2012) also found that the browning phenomenon was more common in the subcutaneous adipose tissue in comparison with visceral adipose tissue in mice. That may be due to the different effects on the regulation of metabolism between subcutaneous and visceral WAT. Visceral WAT was significantly associated with insulin resistance and development of diabetes and metabolic syndrome (Neeland et al., 2013). Conversely, subcutaneous WAT was considered to be beneficial for metabolism (Foster et al., 2013). Overall, SY could reduce the fat mass of HFD-induced obese mice and the mechanism may be involved in promotion of the browning of subcutaneous WAT rather than visceral WAT. Another explanation for the mechanism by which SY reduced the fat mass of HFD-induced obese mice was from our previous study which demonstrated that HSYA, the main effective ingredient of SY, could decrease the lipid accumulation in 3T3-L1adipocytes by promoting the lipolytic-specific enzyme HSL expression. The similar results were obtained both *in vivo* and *vitro* studies which also found that the different concentration of SY (20, 40, and 80 mg kg^−1^) (Zhang et al., 2010) and HSYA (Yu et al., 2011) could decrease the triglyceride contents in HFD-induced hyperlipidemia Wistar rats (Zhang et al., 2010) and steroid-induced (Yu et al., 2011) bone marrow mesenchymal stem cells from Sprague Dawley rats. Therefore, the fat mass reduction in SY treated mice observed in our present study may also be the result of the enhanced lipolysis by promoting the expression of HSL, the key enzyme in the fat metabolism regulation in adipose tissue (Lampidonis et al., 2011).

An animal experiment performed by Zhang et al. (2010) found that SY could reduce the serum TG, TC, LDL-c levels and increase HDL-c level in HFD-induced obese mice. The same phenomenon was also observed in our present study. That may be due to the dependency of long-term intervention which was a characteristic of traditional Chinese medicine. Besides, the difference of drug delivery methods and the sex of mice may be reasons. Zhang et al. (2010) treated the mice with intravenous injection, and half female mice were chosen in their study. Estrogen could improve glucose tolerance, insulin sensitivity (Riant et al., 2009) and reduce TG, TC, LDL-c levels (Homma et al., 2000; Amengual-Cladera et al., 2012) by promoting the activity and expression of lipoprotein lipase with appropriate dose (Pickar et al., 1998). Finally, the increase of UA level was also observed in the SY treated obese mice, but the mechanism is still unclear. Further studies were needed to be done to clarify the possible mechanism in the future.

In summary, SY could significantly reduce the body fat mass and FBG, increase insulin sensitivity of HFD-induced obese mice. The possible mechanism is to promote the browning of subcutaneous WAT and activate the IRS1/AKT/GSK3β insulin signaling pathway in visceral WAT. Our study provides an important experimental evidence for developing SY as a potential anti-obesity and anti-diabetic drug.

## Author contributions

HZ designed the experiments, analyzed data and revised the primary manuscript. XW did the molecular biological experiments, analyzed data and wrote the primary manuscript. HP supervised the experiments. YD did the animal experiments. NL, LW, and HY supervised the experiments, especially the biochemical parameters measurements. FG designed the experiment, supervised the whole experiments and revised the primary manuscript.

## Funding

The study was supported by grants from the National Natural Science Foundation of China (Nos.30600836,81471024 for HZ, Nos.30540036, 30771026, 81370898 for FG), the Natural Science Foundation of Beijing Municipality (No.7082079 for FG), the National Key Program of Clinical Science (WBYZ2011-873 for FG and HZ) and PUMCH Foundation (2013-020 for FG).

### Conflict of interest statement

The authors declare that the research was conducted in the absence of any commercial or financial relationships that could be construed as a potential conflict of interest.

## Abbreviations

SY: safflower yellow
CFDA: China Food and Drug Administration
HSYA: hydroxysafflor yellow A
HSL: hormone sensitive lipase
HFD: high fat diet
WAT: white adipose tissue
IPITT: intraperitoneal insulin tolerance test
IPGTT: intraperitoneal glucose tolerance test
PPIA: Cyclophilin A
HRP: horseradish peroxidase
ECL: enhanced chemiluminescence
SF: standard food
IRS1: insulin receptor substrate 1
GSK3β: glycogen synthase kinase 3β
FOXO1: forkhead box protein O1
PGC1α: peroxisome proliferator-activated receptorgamma coactivator 1α
UCP1: uncoupling protein 1
FNDC5: fibronectin type III domain containing 5
PRDM16: PR domain containing 16
CIDEA, cell death-inducing DNA fragmentation factor: alpha subunit-like effector A
PI3K: phosphatidylinositol 3 kinase.
