# Supplementary material for: The Mechanism by Which Safflower Yellow Decreases Body Fat Mass and Improves Insulin Sensitivity in HFD-Induced Obese Mice
Source: Front Pharmacol. 2016 May 23;7:127. doi: 10.3389/fphar.2016.00127 (PMC4876777; doi:10.3389/fphar.2016.00127)
Supplement: Supplementary file 1 [file Table1.DOCX]

**Table S1. Primers used for RT-qPCR analysis**

| **Genes** | **Sequences (5′-3′)** | **Primers** | **Size (bp)** |
| --- | --- | --- | --- |
| PPIA  NM_008907.1 | 5’-GCTGGACCAAACACAAACGG-3’F  5’-TCCTGGACCCAAAACGCTC-3’R | Forward  Reverse | 139 |
| PGC1α  NM_008904.2 | 5’-TTTACGCAGGTCGAACGAAAC-3’F  5’-GTGGAAGCAGGGTCAAAATCG-3’R | Forward  Reverse | 117 |
| IRS1  NM_010570.4 | 5’-AAGACGCTCCAGTGAGGATT-3’F  5’-AGGAGGATTTGCTGAGGTCATT-3’R | Forward  Reverse | 128 |
| UCP1  NM_009463.3 | 5’-TCTCTGCCAGGACAGTACCCAA-3’F  5’-GAGTCGCAGAAAAGAAGCCACAA-3’R | Forward  Reverse | 108 |
| FNDC5  NM_027402.3 | 5’-TCCTCTCCTCTGCCCCTTAC-3’F  5’-ACAGAGTTCGTTCGCCTTTC-3’R | Forward  Reverse | 122 |
| CIDEA  NM_007702.2 | 5’-GTCAAAGCCACGATGTACGAGA-3’F  5’-TGCAGCATAGGACATAAACCTCAG-3’R | Forward  Reverse | 105 |
| PRDM16  NM_001177995.1 | 5’-GCCGTCCCCACTTCTGTT-3’F  5’-CCTTCATGGCTGCAAAGCTC-3’R | Forward  Reverse | 83 |
| AKT  NM_001165894.1 | 5’-CGGTTCTTTGCCAACATCGT-3’F  5’-AGCTGTGAACTCCTCATCGAA-3’R | Forward  Reverse | 117 |
| FOXO1  NM_019739.3 | 5’-CCTACTTCAAGGATAAGGGCGACA-3’F  5’-AGTTCCTTCATTCTGCACTCG-3’R | Forward  Reverse | 110 |
| GSK3β  NM_019827.6 | 5’-TCCCTCCACATGCTCGGAT-3’F  5’-TTATTGGTCTGTCCACGGTCT-3’R | Forward  Reverse | 97 |
