# Supplementary figures and images for: The Mechanism by Which Safflower Yellow Decreases Body Fat Mass and Improves Insulin Sensitivity in HFD-Induced Obese Mice
Source: Front Pharmacol. 2016 May 23;7:127. doi: 10.3389/fphar.2016.00127 (PMC4876777; doi:10.3389/fphar.2016.00127)

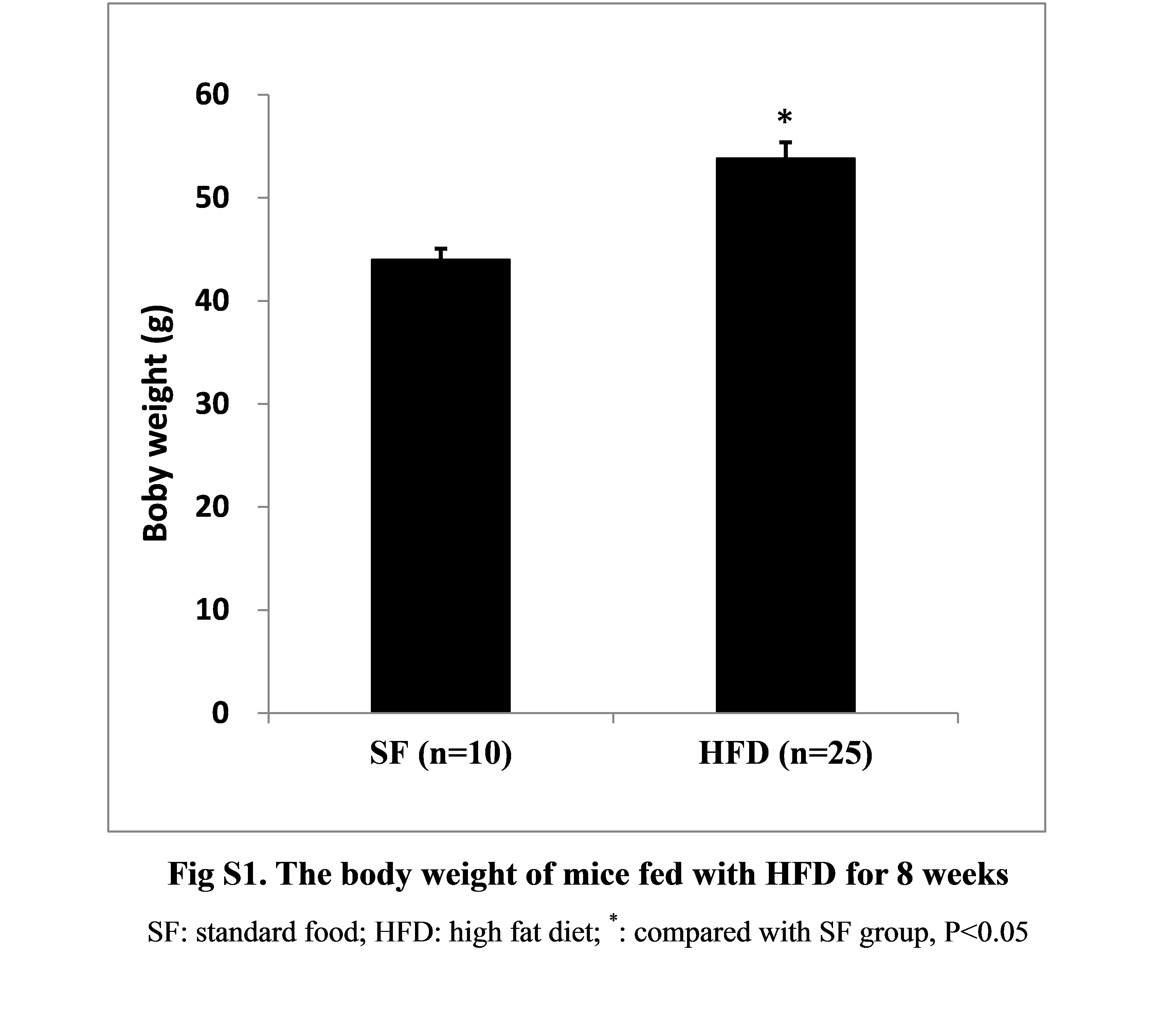

Supplement: Supplementary file 2 [file Image1.JPEG]

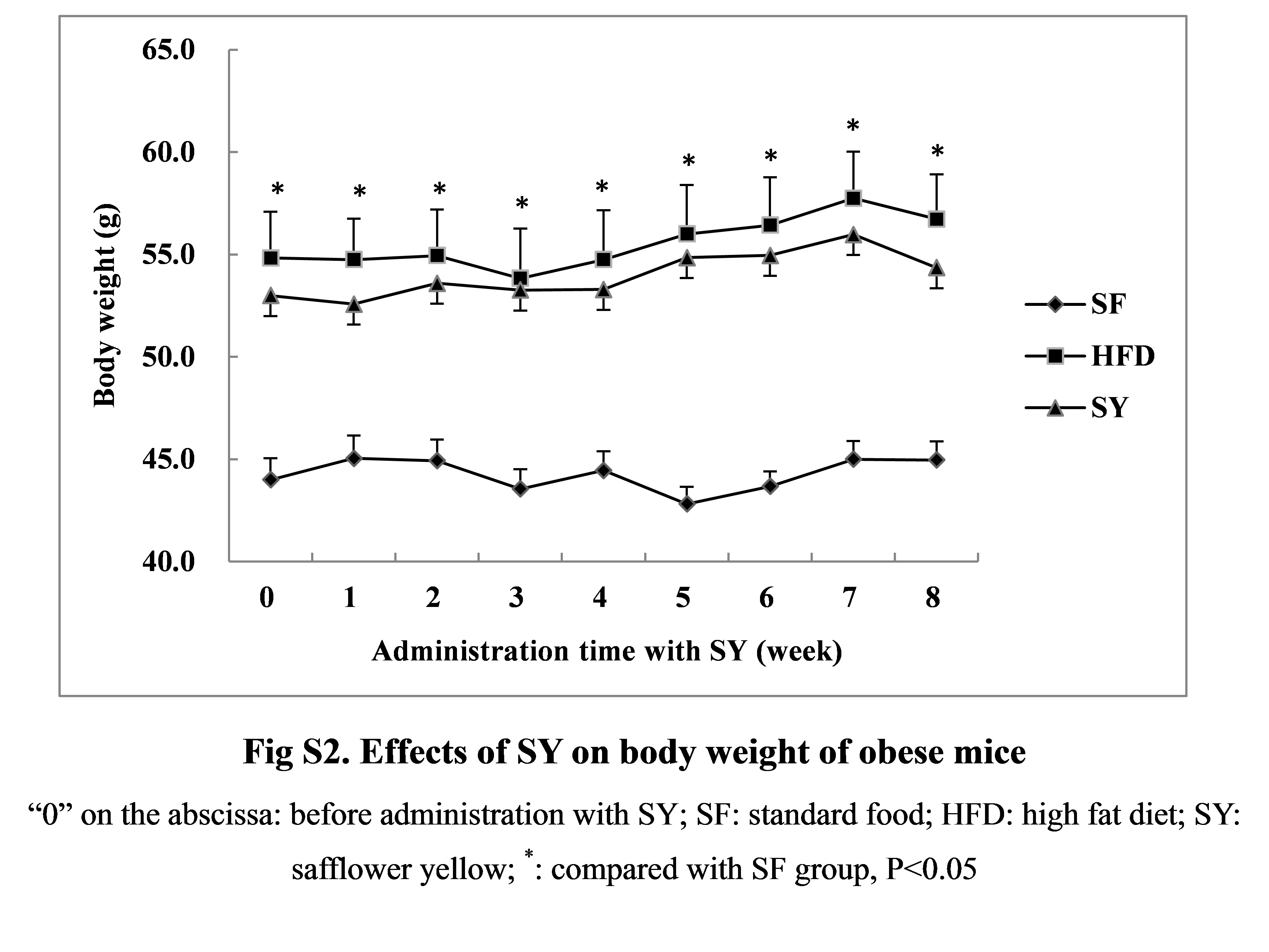

Supplement: Supplementary file 3 [file Image2.JPEG]
